# The herd-immunity threshold must be updated for multi-vaccine strategies and multiple variants

**DOI:** 10.1038/s41598-021-00083-2

**Published:** 2021-11-26

**Authors:** Iraj Yadegari, Mehdi Omidi, Stacey R. Smith

**Affiliations:** 1 Department of Mathematics and Statistics, The University of Ottawa, Ottawa, ON Canada; 2 Department of Mathematics, Ilam University, Ilam, Iran; 3 Faculty of Medicine, The University of Ottawa, Ottawa, ON Canada

**Keywords:** Infectious diseases, Applied mathematics

## Abstract

Several vaccines with different efficacies and effectivenesses are currently being distributed across the world to control the COVID-19 pandemic. Having enough doses from the most efficient vaccines in a short time is not possible for all countries. Hence, policymakers may propose using various combinations of available vaccines to control the pandemic with vaccine-induced herd immunity by vaccinating a fraction of the population. The classic vaccine-induced herd-immunity threshold suggests that we can stop spreading the disease by vaccinating a fraction of the population. However, that classic threshold is defined only for a single vaccine and may be invalid and biased when we have multi-vaccine strategies for a disease or multiple variants, potentially leading policymakers to suboptimal vaccine-allocation policies. Here, we determine which combination of multiple vaccines may lead to herd immunity. We show that simplifying the problem and considering the vaccination of the population as a single-vaccine strategy whose effectiveness is the sample mean of all effectivenesses would not be ideal, because many multi-vaccine strategies with a smaller herd-immunity threshold can be proposed. We show that the herd-immunity threshold may vary due to changes in vaccine-uptake proportions. Moreover, we propose methods to determine the optimal combination of multiple vaccines in order to achieve herd immunity and apply our results to the issue of multiple variants. In addition, we determine a condition for reaching herd immunity in the presence of new emerging variants of concern. We show by example that new variants could influence our estimation of the vaccination reproduction number. It follows that the herd-immunity threshold must be updated not only when multi-vaccine strategies are used but also when multiple variants coexist in the population.

## Introduction

Following the rapid worldwide spread of the coronavirus SARS-CoV-2, which first emerged in late December 2019 in Wuhan, China, a wide range of policies and interventions have been followed by countries in order to respond to the pandemic^1,2^. Multiple waves of the pandemic appeared all around the world, and multiple variants have emerged. Vaccination is one of the main policies to control this worldwide pandemic^3^. However, the vaccine stockpile is not sufficient to vaccinate the entire global population with any single vaccine, so using multiple vaccines is be the best chance to control the spread of the epidemic.

Herd immunity is a crucial determinant for public-health policymakers to contain and potentially eradicate an infectious disease. It plays an important role in controlling the pandemic once a sufficiently high proportion of the population gains immunity through vaccination or infection. The herd-immunity threshold is the proportion of a population that must be vaccinated to stop an infectious disease from spreading. It can be approximated using the basic reproduction number, $\text {R}_0$, which is an epidemic measure used to describe the contagiousness of infectious diseases. It is defined as average number of new infections caused by a single infectious individual in a completely susceptible population^4,5^. Although $\text {R}_0$ is affected by various biological, environmental, sociological and behavioural factors, it is generally reported as a single numeric value with a straightforward interpretation that incidence of the infectious disease is expected to decline if $\text {R}_0$ is less than one and to increase if $\text {R}_0$ is larger than one^5–7^.

Suppose that a specific infectious disease is distributed homogeneously in the population and a single vaccine provides protection against it. Let *p* be the fraction of the susceptible individuals who get vaccinated, with vaccinees selected randomly with the same probability among susceptible individuals. We assume the vaccine provides lifelong immunity with no reinfection and protects only a proportion *E* among those vaccinated. Then the vaccination reproduction number is

1 $$\begin{aligned} \text {R}_V&=\text {R}_0\Big ( 1-p\,E\Big ), \end{aligned}$$

where $E\in (0,1]$ represents the vaccine effectiveness^8,9^. If the vaccine is perfect and provides $100\%$ immunity, then its effectiveness is $E=1$, but if it is imperfect and provides only partial immunity, then $E<1$. The population reaches herd immunity, with incidence of the infection declining, if $\text {R}_V\le 1$, which yields the critical herd-immunity threshold^8^

2 $$\begin{aligned} p^c=\left( 1-\frac{1}{\text {R}_0}\right) \frac{1}{E}\,, \end{aligned}$$

implying that a higher proportion needs to be vaccinated if the vaccine is not perfect^8,9^. Since $p^c$ must belong to the interval [0, 1], we set $p^c$ equal to 1 if the value of (2) is larger than 1. We say a vaccine is effective if $p^c<1$, and herd immunity is thus achievable.

Despite many advanced epidemiological models designed to explain the complex dynamics of infectious diseases^10,11^, Eqs. (1) and (2) are the most applicable formulas in the history of vaccination. While (1) and (2) are based on an assumption of homogeneity of the infection in the population, new results show that (2) represents an upper bound for the herd-immunity threshold under a heterogeneity assumption^9,12^. For example, it has been shown that in age-structured communities with heterogeneous activity groups, the disease-induced herd-immunity threshold would be lower than the one under the homogeneity assumption^9^.

Achieving herd immunity is a multi-dimensional problem, depending not only on an individual’s decision but also the economic, environmental and societal conditions of the population. Considering all effective factors and overparameterising the epidemiological models not only makes them more complex but also makes it hard to extract plausible advice. To prevent overparameterization, we assume that $\text {R}_0$ and *E* are constant and that only a single variant of the virus exists in the population, except in the penultimate section, which discusses a disease with multiple variants. Furthermore, in order to estimate the herd-immunity threshold, we assume that only pharmaceutical intervention is effective and that mass vaccination is the optimal way to control the disease. Henceforth, in this article, achieving herd immunity is equivalent to $\text {R}_V\le 1$.

At the time of writing, multiple vaccines have received approval from governments, and their safety, efficacy, effectiveness and limitations have been analysed^13–16^. There have been remarkable developments in RNA-based technologies to produce new vaccines against infectious diseases. It has been shown that the mRNA vaccines provide a long-lasting immune response and they are potentially much safer than other vaccines. Such vaccines have the potential to be quickly manufactured and to become powerful tools against rapid pandemics. This new technology provides an opportunity to have more than one mRNA vaccine for any vaccine-preventable disease in the future^17,18^. Therefore, extending Eqs. (1) and (2) is essential in order to better understand herd immunity under complex strategies and create a more reliable estimate of the herd-immunity threshold.

Suppose a policymaker is confronted with a pandemic and that multiple vaccines with different effectivenesses $E_1, E_2, \ldots , E_k$ are available to control the spread. Using a single vaccine to vaccinate the entire population may not be the best strategy, because the available vaccine stockpile may be insufficient, and producing additional vaccines takes considerable time. In that situation, the policymaker must allocate different proportions of available vaccines to control the pandemic. However, if we want to use more than one vaccine, each with different effectiveness, for different proportions of the population, then Eqs. (1) and (2) are not valid. Here, we estimate the critical herd-immunity threshold for multi-vaccine allocation strategies. We also propose methods to allocate limited proportions of available vaccines in order to achieve herd immunity.

This article is organised as follows. First, we calculate the critical herd-immunity threshold under multi-vaccine strategies. Next, we discuss different methods to estimate optimal vaccine-allocation strategies. We illustrate our results first for a multi-vaccine strategy for a single strain and secondly for the situation in Canada, with multiple variants and multiple vaccines. Finally, we conclude with a discussion.

## Herd immunity and multiple vaccines

Assume that there are $k>1$ effective vaccines for a single disease and that $E_j$ is the effectiveness of the *j*-th vaccine. Suppose that vaccinees have been selected randomly with the same probability among susceptible individuals. Policymakers can have many strategies to allocate vaccines based on various criteria. Although the most favourable vaccination strategy is using only a single vaccine with the largest effectiveness for the entire population, it is often impossible to receive enough of that vaccine in time, so a combination of different vaccines may be the optimal strategy. Therefore, policymakers have many possible choices to consider various proportions of each available vaccine to achieve herd immunity. We consider the least favourable vaccination strategy to be using only a single vaccine with the smallest effectiveness for the entire population.

**Figure 1:**
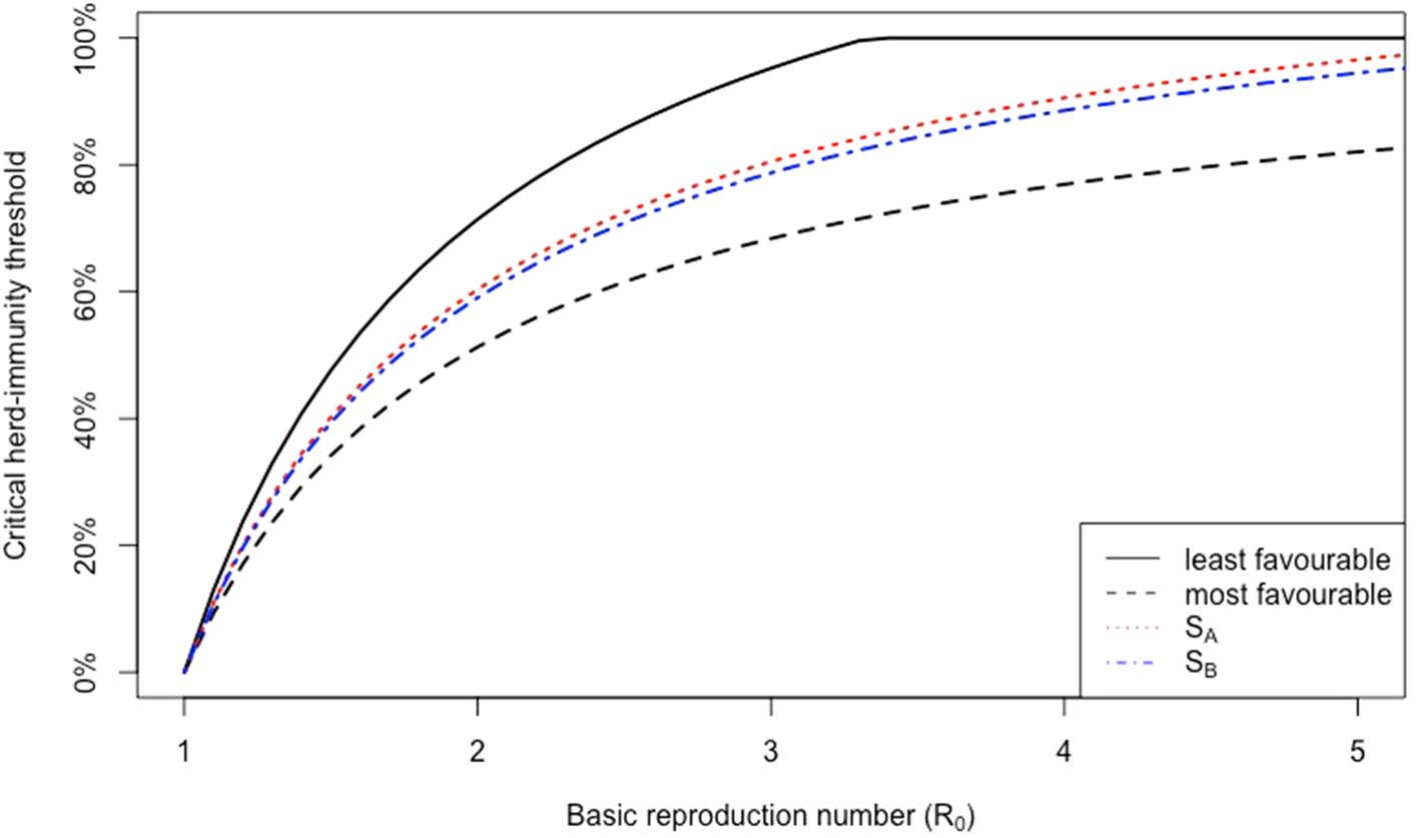
Illustration of the herd-immunity thresholds for four different vaccine-allocation strategies as a function of $\text {R}_0$ when $k=6$ vaccines are available and effectivenesses belong to the interval (0.7, 0.975): the black dashed curve represents the most favourable strategy, and the black solid curve is the least favourable strategy; all vaccine-allocation strategies will be between these two curves; the red dotted curve and the blue dashed-dotted curve are two different examples of vaccines strategies, represented by $\text {S}_A$ and $\text {S}_B$, respectively. The strategy $\text {S}_A$ is equivalent to a single-vaccination strategy whose effectiveness is the average of effectivenesses. We can also propose strategies like $\text {S}_B$ that are closer to the most favourable strategy.

Let $p_j$ be the vaccine-uptake proportion, a fraction of susceptible individuals who receive the *j*-th vaccine, and let $\text {S}$ be an allocation strategy with proportions $(p_1,p_2,\ldots , p_k)'$ such that $\sum _{j=1}^kp_j\le 1$. Furthermore, assume that the susceptible populations receiving different vaccines are disjoint. The vaccination reproduction number with this multi-vaccine strategy is

3 $$\begin{aligned} \text {R}_V^{\text {S}}&=\text {R}_0\Big (1-\sum _{j=1}^k p_jE_j\Big ), \end{aligned}$$

which is the expected reproduction number in the population after vaccination. Our goal is to find a critical lower bound for the total proportion of the population, $p_{_\text {S}}=\sum _{j=1}^k p_j$, that must be vaccinated under the vaccination strategy $\text {S}$ to achieve $\text {R}_V^{S}\le 1$. Obviously, the lower bound of $p_{_\text {S}}$ is between critical herd-immunity thresholds for the least and most favourable strategies; i.e., it belongs to the interval

4 $$\begin{aligned} \Big (\min _j p_j^c\,, \,\max _j p_j^c\Big ), \end{aligned}$$

where $p_j^c$ is the critical herd-immunity threshold if the *j*-th vaccine were the only vaccine used. For any vaccination strategy, the critical threshold would be between the least or most favourable thresholds. The curves in Fig. 1 represent the herd-immunity threshold for different strategies as a function of the basic reproduction numbers when the effectiveness of each vaccines is constant. The black dashed curve and the black solid curve represent the most-favourable and the least-favourable vaccination strategies, respectively. Other strategies’ curves are between these two curves.

**Figure 2:**
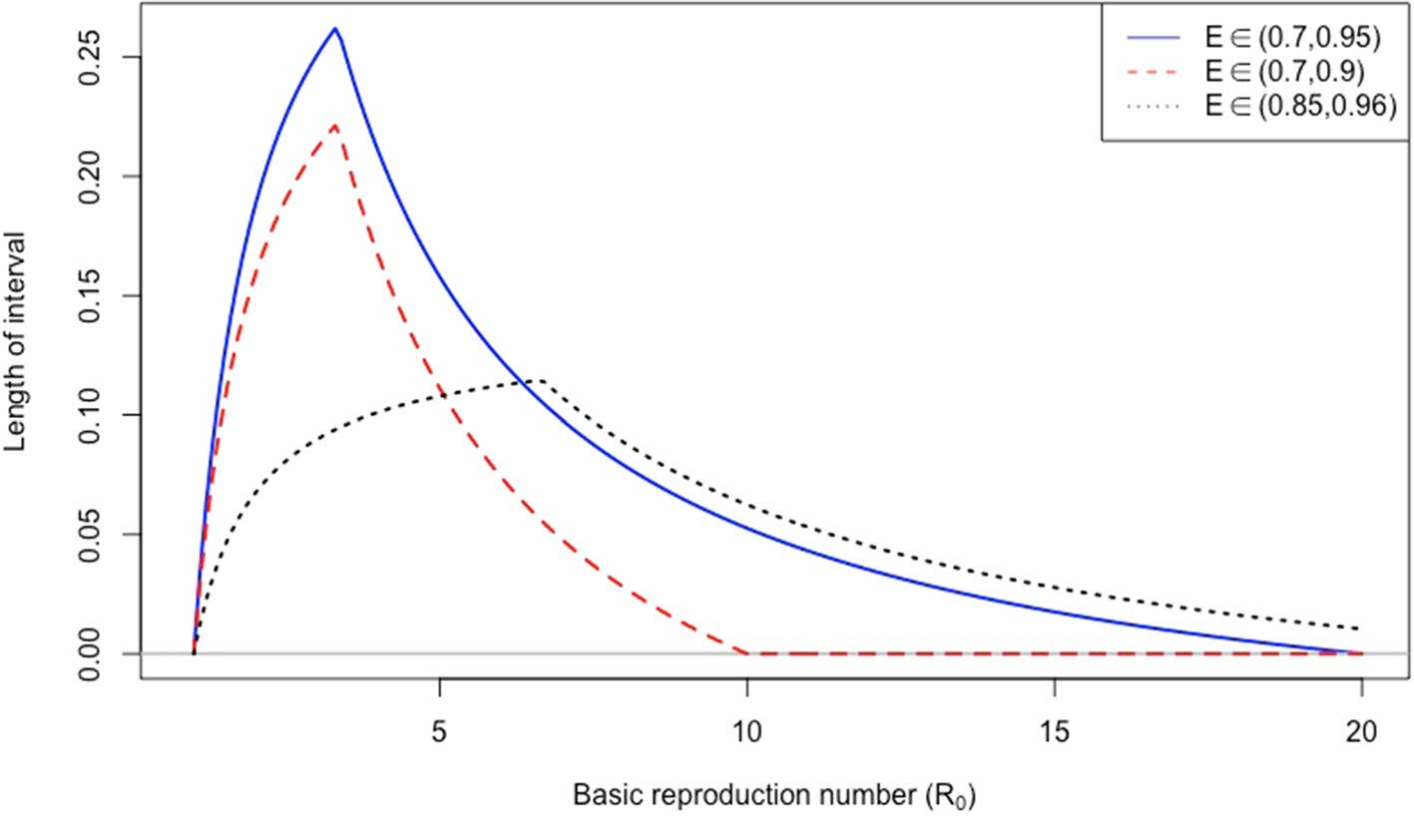
Illustration of the length of the interval (4) as a function of $\text {R}_0$ for three different scenarios: the blue solid curve represents vaccine effectivenesses between 0.7 and 0.95; the red dashed curve represents effectivenesses between 0.7 and 0.9; the black dotted curve represents effectivenesses between 0.85 and 0.96. The first and second scenarios have a peak at the same point with different heights. However, the third scenario has a peak at a larger $\text {R}_0$ value with a smaller height at its peak. Hence the third set of vaccines is effective for a larger range of reproduction numbers, and the difference between the least and the most favourable strategies is smaller than other scenarios.

Consider a set of vaccines that are effective for small values of $\text {R}_0$. Let ${\delta =\max _jp^c_j-\min _jp_j^c}$ be the length of the interval (4), which represents the difference between the least favourable and the most favourable vaccination strategies. Figure 2 illustrates $\delta$ as a function of the basic reproduction number, $\text {R}_0$. Obviously, $\delta$ is increasing for small values of $\text {R}_0$. It is maximised at a point that the least favourable strategy is not effective anymore. The peak of the curve is at the last point of $\text {R}_0$ where all vaccines are effective; i.e., the peak is at $\text {R}_0=1/(1-\min _{j=1}^k E_j)$. After this point, $\delta$ decreases until the most favourable strategy is effective. Then, $\delta$ is minimised to zero at $\text {R}_0=1/(1-\max _{j=1}^k E_j)$, which is the last point that the most favourable strategy is effective. After this point, $\delta$ is constant and equal to zero; i.e., achieving vaccine-induced herd immunity is impossible under any combination of vaccines.

Suppose the policymaker has to select one of the available sets of vaccines for a single infectious disease. Each set defines a different scenario for vaccination and is illustrated by a different curve in Fig. 2. $\delta$ can be interpreted as the effect of changing the share of a vaccine on the herd-immunity threshold. Usually, acceptance rates of vaccines are different, and their shares may change during a vaccination program, which results in a time-dependent and variable herd-immunity threshold. A large value of $\delta$ at the peak means that changing one vaccine’s share may substantially change the herd-immunity threshold. An important point is that the policymakers can propose effective multi-vaccine strategies if the reproduction number is close the peak of $\delta$. On the other hand, improving a vaccination strategy is difficult for values of $\text {R}_0$ when $\delta$ is very small. Hence, replacing a vaccine with one that has a better effectiveness may not have a substantial effect on the herd-immunity threshold. Overall, we can say that the best set of vaccines is the one that takes its peak at a larger value of $\text {R}_0$ and where $\delta$ is small at the peak. An application of this plot is to compare vaccination programs in different countries. Each scenario in Fig. 2 can represent available vaccines for a given country.

### Mean effectiveness of a multi-vaccine strategy

Estimating the smallest proportion of the population that must be vaccinated to achieve $\text {R}_V^{S}\le 1$ is a challenging problem in controlling infectious diseases by vaccination. If we rewrite (3) as $\text {R}_V^{\text {S}}=\text {R}_0\,(1-p_S\bar{E}_S)$, where $p_{_\text {S}}=\sum _{j=1}^k p_j$ is the total proportion of vaccinees, then $\bar{E}_S$ is given by

5 $$\begin{aligned} \bar{E}_{_\text {S}}=\frac{\sum _{j=1}^k p_jE_j}{\sum _{j=1}^k p_j}, \end{aligned}$$

which is a weighted mean of the effectiveness of all vaccines under the vaccination strategy $\text {S}$. $\bar{E}_{_\text {S}}$ measures the mean effectiveness of the vaccination strategy, which is different from the definition of the vaccine effectiveness. When we are dealing with multi-vaccine strategies, it shows how much a combination of vaccines would be efficient. Strategies with greater mean effectiveness are more promising candidates for an efficient impact.

For a single-vaccine strategy, $\bar{E}_{_\text {S}}$ reduces to the effectiveness of a vaccine that is used for whole population. It is maximised for the most favourable and minimised for the least favourable vaccination strategy. When several vaccines are available, considering the vaccination of the population as a single-vaccine strategy whose effectiveness is the sample mean of all effectivenesses is equivalent to rewriting (3) as $\text {R}_V^{\text {S}}=\text {R}_0\,(1-p_S\bar{E})$, where $\sum _{j=1}^kE_j/k$. This in valid only if the same proportion of all vaccines are available, which would not be a realistic assumption during most pandemics.

### Relative effectiveness of a multi-vaccine strategy

In many cases, there are uncertainties in $\text {R}_0$, and having a precise approximation is not possible. Assume that we can represent the uncertainties with an interval, $\text {R}_0\in (a, b)$ such that *a* and *b* are known values. We define a measure of relative effectiveness for a strategy as $\varepsilon ({\mathrm{S}})=1- {\displaystyle {\int }_a^b \mid p^c_{\mathrm {S}}-p^c_{\mathrm {S}_m}\mid d\mathrm {R}_0}/{\displaystyle {\int }_a^b \mid p^c_{\mathrm {S}_\ell }-p^c_{\mathrm {S}_m}\mid d\mathrm {R}_0}$, where $\mathrm {S}_\ell$ and $\mathrm {S}_m$ are the least and most favourable strategies, respectively. If all vaccines are effective for all values of $\mathrm {R}_0\in (a, b)$, then it reduces to $\varepsilon ({\mathrm{S}}) =1-\frac{\mid 1/\bar{E}_{{\mathrm {S}}}-1/\bar{E}_{{\mathrm{S}}_m}\mid }{\mid 1/\bar{E}_{{\mathrm {S}}_\ell }-1/\bar{E}_{{\mathrm{S}}_m}\mid }$. Notice that $\varepsilon (\mathrm {S})\in [0,1]$ and takes its maximum or minimum if the strategy is the most or least favourable strategy, respectively.

### Herd-immunity threshold for a multi-vaccine strategy

Assume that $p_j=\alpha _j\,p_j^c$, where $\alpha _j\ge 0$ is a coefficient and $p_j^c$ is the critical herd-immunity threshold for the *j*-th vaccine if it were used for the whole population. Since the proportions $p_j^c$ are known, given vaccine effectivenesses and the reproduction number of the disease, the vaccination strategy can be determined by $\varvec{\alpha }=(\alpha _1,\ldots ,\alpha _k)'$. (Note that the coefficient of a vaccine is not the fraction of the population who receive that vaccine.) Hereafter, a vaccine’s coefficient is a key concept in the terminology of our discussion. The total proportion of the population that must be vaccinated under the strategy $\mathrm {S}$ can be reformulated as

6 $$\begin{aligned} p^c_{_{\mathrm {S}}}= \left( 1-\frac{1}{\mathrm {R}_0}\right) \, \frac{\sum _{j=1}^k {\alpha _j}/{E_j}}{\sum _{j=1}^k\alpha _j}, \end{aligned}$$

where (6) is a generalization of (2) and includes (2) as a special case if only a single vaccine exists. Furthermore, vaccine-induced herd immunity may be achieved if

7 $$\begin{aligned} \sum _{j=1}^k\alpha _j\ge 1, \end{aligned}$$

or equivalently, if $\mathrm {R}_V\le 1$.

The interpretation of the coefficients may be summarised as follows. In the absence of deriving a closed formula for the herd-immunity threshold from the equation $R_v=R_0(1-\sum _j p_j E_j)$, we formulate the problem using $p_j=\alpha _j p^c_j=\alpha _j (1-1/R_0) /E_j$, or equivalently $\alpha _j=p_j/p_j^c$ (where $p_j^c$ is a function of known values $R_0$ and $E_j$). This formulation leads to the condition $\sum _j\alpha _j\ge 1$ for achieving the herd immunity. Hence $p_j$ is the weight of the *j*-th vaccine with corresponding coefficient $\alpha _j$.

### When a vaccination plan changes

When a vaccination process starts, it can be stopped at any time because of new evidence on the vaccine’s side effects or any other reasons. Many social, political and economical factors may have substantial effects on vaccination progress. Assume that a vaccination process started with *k* vaccines under a strategy $\mathrm {S}_{(1)}$ with proportions $(p_{11}^*,\ldots ,p_{1k}^*)'$, but after a while it has stopped. After or without a pause, the vaccination process will continue with adding, removing or replacing some vaccines. Assume that the second step of the vaccination contains $k'$ vaccines with effectivenesses $E_{21},\ldots , E_{2k'}$ under a new strategy $\mathrm {S}_{(2)}$ with proportions $(p_{21},\ldots , p_{2k'})'$. The vaccination reproduction number is $\mathrm {R}_V^{\mathrm {S}_{(2)}} =\mathrm {R}_0\,\big (1-q-\sum _{j=1}^{k'}p_{2j}E_{2j}\big )$, where $q=\sum _{j=1}^kp_{1j}^*E_{1j}$ is an approximation of the proportion of individuals who are immunised by vaccination in the first step. Note that *q* can be considered as a proportion of individuals who are immunised naturally by infection if the disease provides long-term immunity and if people who are infected are excluded from the vaccination program. Setting $p_{2j}=\alpha _{2j}\,p^c_{2j}$ implies that herd immunity can be reached if

8 $$\begin{aligned} \sum _{j=1}^{k'}\alpha _{j2}\ge 1- \frac{q}{1-\frac{1}{\mathrm {R}_0}}, \end{aligned}$$

which leads to a smaller level of vaccination coverage in order to achieve herd immunity. Note that this result can be extended to cases where the vaccination process has more than two steps.

## Strategy estimation

When an infection exists in the population and vaccination is the main tool to stop spreading it, any failure to stockpile sufficient doses of the vaccine can have drastic consequences on public health. Hence, finding an optimal vaccination strategy that leads to herd immunity is an important step in fighting the disease. In this section, we introduce methods to estimate the unknown vaccine coefficients $\alpha _j$ for $j=1,\ldots , k$ such that *k* is a known integer and $E_1, \ldots , E_k$ are known effectivenesses of available vaccines.

### Ranking-based method

Assigning coefficients to vaccines, calculated with respect to some objective criteria, is a simple way to estimate the optimal vaccination strategies. Let *u* be a positive variable that represents the utility of the vaccines, such that if $u_i<u_j$, then the *j*-th vaccine must have a larger coefficient than the *i*-th vaccine in a vaccination strategy. With this ordering, we can define

9 $$\begin{aligned} \alpha _j= \frac{u_j^n}{\sum _{j=1}^ku_j^n}, \end{aligned}$$

where *n* is a shrinkage parameter that takes non-negative values. By controlling *n*, some coefficients can be shrunk to zero, and their corresponding vaccines are removed from the vaccination program if desired. Here, we introduce some ranking-based methods:

(A) When there is no prior information to sort available vaccines, a simple strategy with equal coefficients for all vaccines can be used; i.e., $\alpha _j= {1}/{k}$. This strategy can be used when there is no meaningful difference between vaccines with respect to decision components. This strategy is equivalent to the case where we simply apply a classic single-vaccine strategy whose effectiveness is the average of the effectivenesses of all the available vaccines.
(B) When effectiveness is the most important criteria to sort vaccines, we may set $u_j=E_j$. This strategy may be used for the case that all vaccines are effective but variation between their effectivenesses is large.
(C) If reducing side effects of the vaccines is the most important criteria, we must maximise the proportion of individuals who remain both unvaccinated and uninfected. This proportion is known as herd effect^19^. In this situation, we can set $u_j=1-p_j^c$ where $p_j^c$ is the herd-immunity threshold for the *j*-th vaccine. Under this strategy, if a vaccine is not effective and we cannot stop the spread of the disease by using only that specific vaccine, its coefficient will be zero.
(D) A strategy can be based on some criteria such as the length of vaccine-induced immunity, vaccine availability, vaccination cost, etc. If we assume that vaccines are ranked based on multiple criteria where $r_{j}$ is the rank of the *j*-th vaccine such that larger ranks are more important than smaller ones, then we can set $u_j=r_j$. This strategy would be the best choice when many factors must be considered to order the vaccines.

### Optimising herd effect and vaccination cost

Usually vaccinating the entire population is impossible, since immunocompromised people, pregnant women or young children often cannot be vaccinated. Therefore, the ideal vaccination policy would be the one with the lowest herd-immunity threshold, which allows the most people to avoid the infection without vaccination. Optimisation techniques can be used to estimate the optimal strategy with the appropriate proportion of vaccines, maximising the benefits while minimising the costs. Optimal vaccination strategies to fight against specific disease for a single vaccine have been discussed^20,21^. Let $\mathrm {S}$ be a vaccination strategy with unknown coefficients $\varvec{\alpha }=(\alpha _1,\ldots , \alpha _k)'$ such that $\sum _{j=1}^k\alpha _j\ge 1$. A reasonable criterion for determining the optimal vaccine-allocation strategy is achieving herd immunity such that the number of individuals who escape the infection without vaccination is maximised, which indirectly decreases the side effects of the vaccine. This is equivalent to minimising $\sum _{j=1}^k {\alpha _j}/{E_j}$, such that $\sum _{j=1}^k\alpha _j\ge 1$.

Optimising the total cost of vaccination is an important consideration for policymakers. Vaccination can have a major effect on developing countries, but health budgets are often limited. Therefore, cost effectiveness is a necessary part of mathematical modelling of the vaccination process. Let *N* be the size of the population of interest, $c_j$ be the cost of the *j*-th vaccine per individual, for $j=1, \ldots , k$. Then a lower bound for the total cost of vaccination under the vaccination policy $\mathrm {S}$ is $N(1-\frac{1}{\mathrm {R}_0})\sum _{j=1}^k\alpha _j {c_j}/{E_j}$. Assuming *N* and $\mathrm {R}_0$ are constant, one can estimate the vaccine weights by minimising the cost and maximizing the herd effect simultaneously, which yields the convex optimisation problem

10 $$\begin{aligned} \min _{\varvec{\alpha }} \sum _{j=1}^k\alpha _j \left( \frac{c_j+1}{E_j}\right) ~~~\text{ such } \text{ that }~~~\sum _{j=1}^k\alpha _j\ge 1, \end{aligned}$$

where all $c_j$ and $E_j$ are known constant values.

#### *Example 1*

Consider a situation of an infectious disease spreading in the population. Here, we explain how our proposed method can be used to allocate specific proportion of available vaccines to reach the herd immunity.

Assume that $\mathrm {R}_0=2.5$ and six vaccines $V_1,\ldots , V_6$ are available with different efficiencies $E_1=0.80$, $E_2=0.85$, $E_3=0.90$, $E_4=0.94$, $E_5=0.95$, and $E_6=0.95$; also, assume $\mathrm {S}_{V_j}$ represents a single-vaccine strategy such that only the *j*-th vaccine is used for the entire population; $\mathrm {S}_{A}^n$, $\mathrm {S}_{B}^n$, $\mathrm {S}_{C}^n$, and $\mathrm {S}_{D}^n$ are multi-vaccine strategies based on a ranking-based method, such that *n* can take values 5, 10 or 20. For the rank-based method, vaccines are ranked based on ascending order of their effectivenesses such that $r_i=i$ and the average of orders is used when ties exist and some effectivenesses are equal. All the information is presented in Table 1. The last three columns of the table represent $p^c_{\mathrm {S}}$, the total proportion of individuals who must be vaccinated under each strategy, relative effectiveness of strategies, $\varepsilon (\mathrm {S})$, and mean effectiveness of strategies, $\bar{E}_{\mathrm {S}}$, respectively. The table body elements are $\alpha _j$ for $j=1,\ldots , 6$.

Table 1 is divided into two parts: the first represents single-variance strategies, while the second describes multi-vaccine strategies. The first part consists of six single-vaccine strategies such that $p_j^c=P_{\mathrm {S}_{V_j}}^c$ for $j=1, \ldots , 6$. Therefore, $p_1^c=0.75$, $p_2^c=0.706$, $p_3^c=0.667$, $p_4^c=0.638$, $p_5^c=0.632$ and $p_6^c=0.632$ are critical vaccine thresholds for $V_1,\ldots , V_6$, respectively. The least favourable strategy $\mathrm {S}_{V_1}$ implies that at least $75\%$ of the population must be vaccinated while the most favourable strategy needs only $63.2\%$ of the population to be vaccinated.

Note that an infinite number of multi-vaccine strategies can be proposed. However, only a few strategies are discussed, based on estimation methods described in the ranking-based method. There are four categories of multi-vaccine strategies in the second part of Table 1:

**Table 1:** Illustration of some strategies with their specific weights, herd-immunity threshold, relative effectiveness and mean effectiveness of strategies.

	Strategy	Vaccines (effectiveness)						$p^c_{\mathrm {S}}$	$\varepsilon (\mathrm {S})$	$\bar{E}_{\mathrm {S}}$
		$V_1(0.80)$	$V_2(0.85)$	$V_3(0.90)$	$V_4(0.94)$	$V_5(0.95)$	$V_6(0.95)$			
Single-vaccine strategies	$\mathrm {S}_{V_1}$	1	0	0	0	0	0	0.750	0.000	0.80
	$\mathrm {S}_{V_2}$	0	1	0	0	0	0	0.706	0.373	0.85
	$\mathrm {S}_{V_3}$	0	0	1	0	0	0	0.667	0.704	0.90
	$\mathrm {S}_{V_4}$	0	0	0	1	0	0	0.638	0.943	0.94
	$\mathrm {S}_{V_5}$	0	0	0	0	1	0	0.632	1.000	0.95
	$\mathrm {S}_{V_6}$	0	0	0	0	0	1	0.632	1.000	0.95
Multi-vaccine strategies	$\mathrm {S}_A$	1/6	1/6	1/6	1/6	1/6	1/6	0.671	0.672	0.895
	$\mathrm {S}_B^1$	0.13	0.15	0.17	0.18	0.19	0.19	0.667	0.691	0.898
	$\mathrm {S}_B^{10}$	0.04	0.08	0.15	0.23	0.25	0.25	0.650	0.850	0.924
	$\mathrm {S}_B^{20}$	0.01	0.03	0.10	0.25	0.30	0.30	0.640	0.926	0.937
	$\mathrm {S}_C^1$	0.12	0.15	0.17	0.18	0.19	0.19	0.665	0.722	0.903
	$\mathrm {S}_C^{10}$	0.01	0.03	0.11	0.25	0.30	0.30	0.640	0.926	0.937
	$\mathrm {S}_C^{20}$	0.00	0.00	0.05	0.25	0.35	0.35	0.635	0.966	0.944
	$\mathrm {S}_D^1$	0.05	0.10	0.14	0.19	0.26	0.26	0.650	0.838	0.922
	$\mathrm {S}_D^{10}$	0.00	0.00	0.00	0.02	0.49	0.49	0.632	1.000	0.950
	$\mathrm {S}_D^{20}$	0.00	0.00	0.00	0.00	0.50	0.50	0.632	1.000	0.950

- The first category of the second part is $\mathrm {S}_A$, where uniform coefficients are assigned to the vaccines. It leads to vaccination coverage equal to 0.67, which is equivalent to vaccinating the entire population with a single vaccine with effectiveness of $E=0.90$. Since $\alpha _j=1/6\approx 0.167$ and $p_j=\alpha _jp^c_j$, then $p_1\approx 0.125$, $p_2\approx 0.118$, $p_3\approx 0.111$, $p_4\approx 0.107$, $p_5\approx 0.106$ and $p_6\approx 0.106$ are proportions of the population that will be vaccinated by $V_1,\ldots , V_6$ to reach herd-immunity.
- The second category is based on effectiveness values. By increasing the shrinkage parameter, the coefficients approaches zero slower than strategies in Categories C and D. For example, when $n=20$, Strategy $\mathrm {S}_B^{20}$ proposes to use all vaccines, while $\mathrm {S}_C^{20}$ and $\mathrm {S}_D^{20}$ remove some vaccines from the vaccination program.
- Category C of multi-vaccine strategies is based on herd immunity and assigns the largest coefficient to a vaccine that lets more people escape the infection without vaccination. Strategies in Category C can be close to some strategy in Category B. For example, strategies $\mathrm {S}_C^{10}$ and $\mathrm {S}_B^{20}$ suggest similar coefficients for vaccines.
- The last category is based on ranking vaccines. The shrinkage parameter reduces the coefficients faster than Categories B and C. For example, $\mathrm {S}_D^{20}$ assigns coefficients only to vaccines with largest effectiveness.
- For a constant value of the shrinkage parameter $n=n_0$, $\mathrm {S}_D^{n_0}$ is better than $\mathrm {S}_C^{n_0}$ and $\mathrm {S}_C^{n_0}$ is better than $\mathrm {S}_B^{n_0}$, according to their mean and relative effectivenesses. When *n* is extremely large, all strategies in Categories B, C and D approach $\mathrm {S}_D^{20}$, which is the most favourable strategy.
- Note that relative effectiveness $\varepsilon (\mathrm {S})$ represents the power of a possible combination of available vaccines with respect to the least and most favourable strategies; its small value does not imply weakness of the strategy to stop the infection. For example, the relative effectiveness is always small for strategies that put large weight on the least favourable vaccine regardless of its effectiveness. Hence, both mean and relative effectivenesses together show the power of a multi-vaccine strategy to contain the infection.

Making decisions for vaccine allocation is a complex problem and depends on multi-factor priorities. Table 1 proposes plausible strategies that satisfy logistic, financial and social conditions of the public-health system. Overall, all strategies in Categories B, C, and D are better than $\mathrm {S}_A$, which is equivalent to a single-vaccine strategy whose effectiveness is the sample mean of all effectivenesses.

## When several variants exist

When several variants of a virus exist in the population, some adjustments are needed to estimate the herd-immunity threshold. Assume that only a single vaccine exists to control spreading a virus with *m* variants and that $\pi _1,\ldots , \pi _m$ are the proportions of each variant, such that $\sum _{j=1}^m \pi _j=1$. In addition, $E_1, \ldots , E_m$ are effectivenesses of the vaccine against variants. Under this setting, the vaccination reproduction number is $\bar{{\mathrm{R}}}_V=\bar{{\mathrm{R}}}_0\,(1-p\bar{E})$, where $\bar{\mathrm {R}}_0=\sum_{j=1}^m\pi_j\mathrm{R}_0^j$ is the average reproduction number for all variants and $\bar{E}=\sum _{j=1}^m \pi _j E_j$ is the average effectiveness of the vaccine against the virus in the population. Note that estimating $\bar{E}$ would be a challenging problem if adequate and precise clinical tests to diagnose different variants are not done with respect to spatial distribution of the disease, which could lead to biased estimation of the prevalence of different variants. In addition, distribution of variants is time-dependent; consequently, $\bar{E}$ will change over time.

If *k* vaccines are available for a virus with *m* variants, we assume that $E_{ij}$ is the effectiveness of the *i*-th vaccine against the *j*-th variant. Therefore, the vaccination reproduction number would be $\bar{{\mathrm{R}}}_V=\bar{{\mathrm{R}}}_0\,(1-\sum _{j=1}^k p_i \bar{E}_i)$, where $\bar{E}_i=\sum _{j=1}^m \pi _j E_{ij}$ is the average effectiveness of the *i*-th vaccine against the virus. Herd immunity may thus be achieved if 

11 $$\sum_{j=1}^k\alpha_j\left(\bar E_i\over E_{i1}\right)\geq1,$$

where $p_i=\alpha_i[(1-1/R_0)/E_{i1}]$ and $E_{i1}$ is the effectiveness of the i-th vaccine against the original strain of the virus. This implies that when new emerging variants reduce the effectiveness of the vaccines, more vaccines are needed to reach herd immunity.

### *Example 2*

In this example, we show how to estimate the vaccination reproduction number when several variants with different prevalence fractions exist in the population and multiple vaccines are used to control the spread of the disease. Multiple studies have evaluated the effectiveness of different vaccines against important variants of COVID-19^22^. Real-world data on vaccine effectiveness against all variants recognized by the World Health Organization (WHO) are currently limited. New emerging variants of concern (VOC)—currently including Alpha (B.1.1.7), Beta (B.1.351), Gamma (P.1) and Delta (B.1.617.2)—are more contagious and can potentially increase disease severity and decrease vaccine effectiveness^22–26^.

Vaccination against symptomatic infection of COVID-19 in 2020–2021 has been deployed using BNT162b2 (Pfizer-BioNTech), mRNA-1273 (Moderna) and ChAdOx1 (AstraZeneca) vaccines^26^. The effectiveness of these vaccines against VOC has been estimated by Nasreen et al.^26^. Estimates of the effectiveness of vaccines against infection with partial and full vaccination in Canada is reported in Table 2. However, due to insufficient data for Moderna and AstraZeneca vaccines, effectiveness of fully vaccinated cases was not estimated for these vaccines. Therefore, we estimated missing values and used other sources to provide estimates for data that we need. We make the following observations about Table 2.

- The second column represents the type of vaccine. In some cases, the vaccine type was not reported or individuals used mixed vaccines; i.e., first and second doses were not from the same vaccine.
  The third column presents cumulative percentages of people who had received a COVID-19 vaccine by July 31, 2021. This information is available on website of the government of Canada at https://health-infobase.canada.ca.
  The fourth through seventh columns represent effectiveness of vaccines against the original strain and VOC. Since both Beta and Gamma variants share common mutations^26^ and insufficient numbers of them were sequenced, they are classified together.
- A report from Public Health Ontario, https://www.publichealthontario.ca, provides a list of the reported effectiveness measures for vaccines around the world. In general, vaccine effectiveness for preventing symptomatic infection 3–4 weeks after receiving a single dose is between 60% and 80% for the Pfizer-BioNTech and Moderna, and between 60% and 70% for AstraZeneca. Since the effectiveness of vaccines decreases against VOC, we estimate the effectiveness of Pfizer-BioNTech, Moderna and AstraZeneca for a single dose against the original strain at 80%, 80% and 70%, respectively.
- The Public Health England vaccine effectiveness report from March 2021, https://assets.publishing.service.gov.uk, shows that vaccine effectiveness against infection is about 85%, seven or more days after the second dose.
- Lopez Bernal *et al.* showed that vaccine effectiveness after two doses of the AstraZeneca vaccine is 74% against the alpha variant and 67% against the delta variant^25^. We used this information to estimate the missing values.
- The final column represents average effectiveness of each vaccine against all listed variants, which is calculated as a weighted mean of effectiveness measures; i.e., $\bar{E}_i=\sum _{j}\pi _jE_{ij}$. The weights are prevalence of variants, which is represented in the bottom row of Table 2. This row presents cumulative prevalence of VOC as listed in the Canada mutation report, dated August 15, 2021. It is available at https://outbreak.info, which provides results of screening tests for mutations and whole-genome sequencing to assign COVID-19 lineage. The cumulative prevalence is the ratio of the genetic sequences containing the lineage or mutations to all genetic sequences collected since the identification of lineage or mutations in that location.
- The fourth row of single-dose and two-dose vaccination show “Vaccines not reported”, which represent a group of people who are vaccinated by Pfizer-BioNTech, Moderna or AstraZeneca, but the name of their vaccines are missing from the reported data. These account for 3.33% and 13.2% of people take single-dose and two-dose vaccines, respectively. We have estimated their corresponding rows by a weighted mean of effectiveness of all the vaccines with respect to their cumulative percentages in third column.
- The vaccine effectiveness of Moderna for full vaccination was estimated only against Alpha but was missing for other variants. We used the effectiveness of Moderna after first dose to estimate the missing values.
- Note that only 0.1% and 0.02% of people were partially and fully vaccinated using other vaccines. Therefore, we ignored this category.
- In order not to underestimate the reproduction number of the virus, we estimate missing values with smallest similar data. For example, in full vaccination, effectiveness of AstraZeneca was available only for the original variant, so we estimate its effectiveness against VOC with the same value.
- Mixed vaccination was approved by the government of Canada, and a proportion of people used different vaccines for their first and second doses. Since it was not clear which vaccines were mixed together, we assumed that most of them used AstraZeneca for the first dose and Moderna or Pfizer-BioNTech for the second dose. Therefore, to prevent overestimating the missing effectiveness values, we estimated the effectiveness of the mixed vaccines with the smallest effectiveness of those three vaccines for full vaccination.
- Empirical research shows substantial increases in estimated reproduction numbers of VOC, compared to the original strain. These increases are 29%, 25%, 38% and 97%, respectively, for Alpha, Beta, Gamma and Delta variants. We considered an average of 30% increase for Beta/Gamma^27^.

**Table 2:** Cumulative percentage of people who have received a COVID-19 vaccine in Canada by vaccine product and effectiveness in partially and fully vaccinated cases as of July 31, 2021.

	Vaccine type	Percentage ($p_i$), %	Effectiveness against variants ($E_{ij}$)				$\bar{E}_i$, %
			Original strain, %	Alpha, %	Beta/gamma, %	Delta, %	
Partial (single-dose) vaccination	Pfizer	5.40	80	66	60	56	71
	Moderna	2.20	80	83	77	72	80
	AstraZeneca	0.12	70	64	48	67	65
	Vaccine not reported	3.33	92	71	65	61	74
Full (two-doses) vaccination	Pfizer	28.12	95	89	84	87	91
	Moderna	8.01	94	92	77	72	89
	AstraZeneca	0.56	85	74	48	67	75
	Vaccine not reported	13.2	95	89	82	83	90
	mixed	9.7	85	74	48	67	75
	Variant prevalence ($\pi _j$)	–	47	31	15	6	–

We can thus estimate the vaccination reproduction number using our proposed method. If we set $\mathrm {R}_0=2.5$ for the original strain, we have ${\mathrm{R}}_V = {\mathrm{R}}_0\, ( 1-\sum _{_{Partial}} p_i E_{i1} -\sum _{_{Full}} p_i E_{i1} )=0.8817$ when only the original variant exists in the population. However, if several variants coexist and we modify the reproduction number with respect to their prevalence distribution and effectiveness of vaccines against them according to Table 2, then $\bar{{\mathrm{R}}}_V=\bar{{\mathrm{R}}}_0\, ( 1-\sum _{_{Partial}} p_i \bar{E}_{i} -\sum _{_{Full}} p_i \bar{E}_{i} )=1.17$ is our modified reproduction number. It follows that $\bar{\mathrm {R}}_V/ {\mathrm {R}}_V\approx 1.32$; hence if we do not modify our calculations in presence of new emerging variants that are potentially resistant against vaccines, we would underestimate the reproduction number, which may lead us to biased decisions. Note that $\bar{\mathrm {R}}_V/ \mathrm {R}_V$ may change substantially if the prevalence of new emerging vaccine-resistant variants increases. This example illustrates how multiple variants may affect the vaccination reproduction number.

## Discussion

We have analysed the complexity of herd immunity in the presence of multiple vaccines and multiple variants and proposed an update to the herd-immunity threshold when multiple vaccines are used or when several variants of the virus coexist. This update considers not only distribution of the vaccines but also the prevalence of variants and effectiveness of vaccines against them. Our contributions bring several interesting insights to the literature of herd immunity and vaccine-allocation strategy. First, we calculated the herd-immunity threshold for combinations of multiple vaccines and showed that it depends on not only the reproduction number and vaccine effectiveness but also the proportion of vaccinees for all vaccines. Moreover, we determined which combination of vaccines would lead to herd immunity. Second, we showed that when several vaccines are available, simplifying the problem and considering the vaccination of the population as a single-vaccine strategy whose effectiveness is the sample mean of all effectivenesses, $\sum _{j=1}^kE_j/k$, is not ideal, because many multi-vaccine strategies with smaller herd-immunity thresholds can be proposed. Third, our proposed modification of the herd-immunity threshold enables us to search for optimal combination of vaccines in order to reach herd immunity. We described methods to search for optimal vaccine-allocation strategies based on different priorities. Fourth, we showed that vaccine-allocation policies are equivalent to simple convex optimisation problems if we wish to maximise the herd effect and/or minimise the cost of a vaccination strategy. Fifth, our definition of the mean effectiveness, which is a novel measure for describing efficiency of a vaccination program, can be used to compare different vaccination strategies. Finally, we extended the main results of this work to the situation where multiple variants of concern exist in the population. We determined conditions for reaching herd immunity in the presence of new emerging variants of concern. We have shown that several coexisting variants may change the vaccination effectiveness and have illustrated it with a Canadian example. It follows that the herd-immunity threshold must be updated with respect to the prevalence distribution of variants and effectiveness of vaccines against them.

Vaccine hesitancy is one of the most important problems in achieving herd immunity. The WHO has listed vaccine hesitancy as one of ten threats to global health. Vaccine hesitancy is defined as a delay in acceptance or refusal of vaccines despite the availability of vaccination services^28^. Recent investigations report different vaccine-acceptance rates for available vaccines for COVID-19^29,30^. Therefore, vaccine-acceptance rates or vaccine-hesitancy rates are potential components that can be used to optimise the vaccine’s coefficients in order to introduce an optimal vaccination strategy that minimises overall vaccine hesitancy.

Having more vaccines with large efficacies increases the strength of any vaccination program. However, the speed of vaccination should not be underrated in pandemic management. Containing the disease in the shortest possible time is an ideal goal, which needs to use the maximum amount of available vaccines. Therefore, having more available vaccines would be considered a component of an optimal strategy.

If we assume that an individual can be immunised after infection for a short period of time, then the vaccination reproduction number is time-dependent and can be modified as ${\mathrm{R}}_V(t) = {\mathrm{R}}_0(1-q(t)-\sum_{j=1}^kp_jE_j)$, where $q(t)$ is the proportion of the population that is still immunised naturally at time *t*, which can be estimated empirically from antibody test results or from related epidemiological models. This implies that, with natural immunisation after infection, herd immunity may be achieved faster than predicted by a vaccine-induced herd-immunity threshold. In contrast, we have shown that when multiple variants of the virus are emerging in the population, more vaccines are needed to reach the herd-immunity level. Therefore, a generalised condition for reaching herd immunity is $\sum_{j=1}^k\alpha_j(\bar E_i/E_{i1})\geq1-q(t)/(1-1/\mathrm{R}_0)$, which includes (8) and (11) as special cases. Hence it is reasonable to expect to reach herd immunity at larger vaccination levels when the probability of reinfection is significantly large.

Our results have some limitations, which should be acknowledged. The first is that there is no measurement error in the estimates of R_0_ or *E*. the second is that they are constant across all communities in the population, which implies that the population is homogeneous; this is not true in age-structured populations, for instance. The third is that they are not time-dependent. Further work is required to generalise our results in these circumstances.

Vaccination at a level larger than the herd-immunity threshold does not imply that the infection can be stopped if vaccination has a different pattern from the infection. For example, a vaccination policy based on age prioritisation can save more lives if age is the only heterogeneous characteristic of the population and infection is uniformly distributed spatially. Without considering the spatial distribution of the infection, the vaccination process can waste valuable time and cause the disease to spread faster in the population. In an effective vaccination policy, spatial hotspots must take priority, and vaccines must therefore be distributed with the same spatial pattern as the infection itself. Only if proactive measures are taken will we be able to prevent the most infections and save the most lives using all possible vaccines at our disposal.
